# Traumatic Brain Injury and Neuronal Functionality Changes in Sensory Cortex

**DOI:** 10.3389/fnsys.2016.00047

**Published:** 2016-06-02

**Authors:** Simone F. Carron, Dasuni S. Alwis, Ramesh Rajan

**Affiliations:** ^1^Neuroscience Research Program, Biomedicine Discovery Institute, Department of Physiology, Monash UniversityMonash, VIC, Australia; ^2^Ear Sciences Institute of AustraliaPerth, WA, Australia

**Keywords:** TBI, brain injury, sensory cortex, neuronal encoding, inhibition, prognosis

## Abstract

Traumatic brain injury (TBI), caused by direct blows to the head or inertial forces during relative head-brain movement, can result in long-lasting cognitive and motor deficits which can be particularly consequential when they occur in young people with a long life ahead. Much is known of the molecular and anatomical changes produced in TBI but much less is known of the consequences of these changes to neuronal functionality, especially in the cortex. Given that much of our interior and exterior lives are dependent on responsiveness to information from and about the world around us, we have hypothesized that a significant contributor to the cognitive and motor deficits seen after TBI could be changes in sensory processing. To explore this hypothesis, and to develop a model test system of the changes in neuronal functionality caused by TBI, we have examined neuronal encoding of simple and complex sensory input in the rat’s exploratory and discriminative tactile system, the large face macrovibrissae, which feeds to the so-called “barrel cortex” of somatosensory cortex. In this review we describe the short-term and long-term changes in the barrel cortex encoding of whisker motion modeling naturalistic whisker movement undertaken by rats engaged in a variety of tasks. We demonstrate that the most common form of TBI results in persistent neuronal hyperexcitation specifically in the upper cortical layers, likely due to changes in inhibition. We describe the types of cortical inhibitory neurons and their roles and how selective effects on some of these could produce the particular forms of neuronal encoding changes described in TBI, and then generalize to compare the effects on inhibition seen in other forms of brain injury. From these findings we make specific predictions as to how non-invasive extra-cranial electrophysiology can be used to provide the high-precision information needed to monitor and understand the temporal evolution of changes in neuronal functionality in humans suffering TBI. Such detailed understanding of the specific changes in an individual patient’s cortex can allow for treatment to be tailored to the neuronal changes in that particular patient’s brain in TBI, a precision that is currently unavailable with any technique.

## The Clinical Problem of Traumatic Brain Injury (TBI)

Traumatic brain injury (TBI) is caused by direct blows to the head or inertial forces during relative head-brain movement. TBI can result from physical trauma to the head, in sports accidents, physical abuse, motor vehicle accidents, military conflict and terrorist activity. The first three account for most TBI in civilians and the latter two for a large increase in TBI in defense personnel and civilians (Narayan et al., 2002; Werner and Engelhard, 2007; Myburgh et al., 2008; Park et al., 2008; Risdall and Menon, 2011). TBI is a major global health issue with an incidence of at least 200/100,000 population and mortality rate for severe TBI of 20–30% in developed countries and up to 90% elsewhere (VNI, 2007/2008; Helps et al., 2008; Faul et al., 2010; Risdall and Menon, 2011). An estimated 52,000 people with TBI die annually in the USA, with TBI being a contributing factor in up to 30.5% of all injury-related deaths (Faul et al., 2010). It is a continuing problem for victims, families and the community since even mild TBI may result in life-long disability, with enormous social and medical burdens; total life-time expenses in moderate-to-severe TBI were estimated to be $8.6 billion in 2008 in Australia alone (Collie et al., 2010) and $60 billion inclusive of direct and indirect medical costs in the USA in 2000 (Corso et al., 2006). Even mild TBI is associated with high rates of cognitive impairment, often affecting young people with a long life ahead.

Current treatment options in TBI are scarce and of limited effectiveness. To date there has been no successful Phase III clinical trial of a therapy and there are no FDA-approved therapies for mitigating the effects of TBI. In particular, with respect to drug therapies, in December 2014 the PROTECT III Phase III Randomized Controlled Trials (RCT) of progesterone treatment for TBI report noted “more than 30 clinical trials have investigated various compounds for the treatment of acute TBI, yet no treatment has succeeded at the confirmatory trial stage” (Wright et al., 2014). These past translational failures may have arisen from not having sufficient fine grain detail of the different pathophysiologies or differential prognosis in different injury models—likely because of not using techniques that provide fine grain resolution of brain activity changes in different forms of TBI. We argue here that electrophysiological monitoring of neuronal activity, especially cortical neuronal activity, provides a mechanism to do so individually in TBI patients. To establish this point we will review the understanding that has been gained from use of systems neuroscience techniques to study the effects of TBI on cortical neuronal functionality.

This review is couched in the context that much is known of molecular and anatomical changes post-TBI but, until recently, almost nothing was known of the changes in cortical neuronal function and information processing that cause long-term cognitive, motor and sensory deficits and their evolution over time. Given that effective connectivity and optimal network structure is essential for proper information processing in the brain, and functional brain abnormalities are associated with pathology in connectivity and network structures, people with mild to moderate diffuse TBI can recover simple motor skills such as grip strength and finger tapping (Haaland et al., 1994) in the first year after trauma. Even after severe forms of TBI motor impairment show a pattern of improvement (Walker and Pickett, 2007) possibly due to development of compensatory behaviors and uninjured intact motor areas providing recovery via intracortical connectivity with other cortical regions and/or through their direct corticospinal projection pathways (Nudo, 2013), but have prolonged cognitive deficits (Strich, 1956; Bawden et al., 1985; Povlishock, 1993; Haaland et al., 1994; Gagnon et al., 1998; Graham et al., 2000; Werner and Engelhard, 2007; Draper and Ponsford, 2008; Little et al., 2010; Fozouni et al., 2013; Bayley et al., 2014). Cognition encompasses abilities such as attention, memory, language, reasoning and problem solving, and is essential for almost every aspect of our lives. It is impacted after even mild TBI, with impairment in memory, speed of information processing and executive functioning being strongly associated with the degree of functional outcome following injury, affecting many areas including employment, education, and social participation (Strich, 1956; Bawden et al., 1985; Haaland et al., 1994; Gagnon et al., 1998; Graham et al., 2000; Draper and Ponsford, 2008; Little et al., 2010). Thus the cognitive deficits caused even by mild TBI have wide ranging effects on every facet of a patient’s life.

Commonly-used methods such as behavioral assessment and structural neuroimaging do not allow for precise monitoring of the evolution of TBI-induced changes in neuronal functionality. Only electrophysiology can provide the high-precision information needed to monitor and understand the temporal evolution of neuronal functionality changes. We have used this technique in rats to detail changes in cortical neural function at different time points after either closed skull TBI or open skull TBI (Alwis et al., 2012, 2013; Johnstone et al., 2013, 2014, 2015; Yan et al., 2013). Our studies in animal models of closed-skull and open-skull TBI have now provided compelling evidence that TBI alters sensory cortical neuronal functionality (Alwis et al., 2012, 2013; Johnstone et al., 2013, 2014, 2015; Yan et al., 2013). We will review these and other relevant studies in a systems neuroscience framework after a brief review of neuronal changes in TBI.

## A Brief Overview of TBI Effects on Brain Neurons

**Figure 1:**
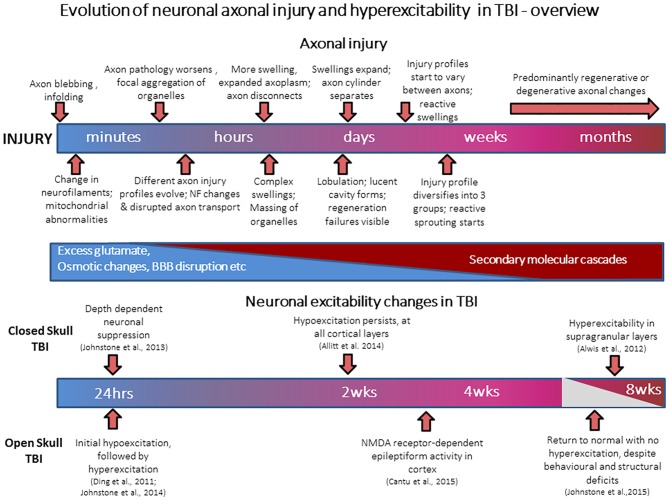
**Evolution of neuronal axonal injury and hyperexcitability after traumatic brain injury (TBI).** The schematic figure summarizes events occurring during the progression of axonal injury from acute post-injury periods (minutes to hours), through to late post-injury periods (weeks to months). Also presented is a summary of known electrophysiological changes in neuronal excitability in cortex after TBI, and the time-points at which they occur.

During head trauma, inertial forces from rapid acceleration-deceleration and rotation of the brain lead to diffuse TBI, where there is diffuse axonal injury (DAI); tearing injuries to axons, causing axonal swelling and disconnection (Povlishock, 1993; Gaetz, 2004; Werner and Engelhard, 2007). Many molecular cascades are activated to cause secondary brain damage via multi-factorial processes including oxidative stress, excitotoxicity, hypoxia-ischemia, inflammation and edema, leading to continuous changes in axon pathology over minutes-to-weeks (Povlishock, 1993; Gaetz, 2004; Werner and Engelhard, 2007). The molecular and anatomical changes in TBI follow a complex time course, and neuron pathology evolves from minutes to weeks post-trauma, as shown in the simplified summary of Figure 1. Very severe head injury causes lesions (focal TBI) visible on standard imaging but diffuse TBI is believed to be underdiagnosed as it is not visible with CT or MRI [now there is some success with diffusion tensor imaging (DTI), for white matter injury (Fozouni et al., 2013)]. Focal injury includes contusions, which are usually superficial bruises of the brain affecting the cortex and in more severe cases the underlying white matter itself. About 70–75% of human TBI cases show diffuse TBI and ~20–25% suffer focal and diffuse injury. The fluid percussion (FP) and controlled cortical impact (CCI) models are representative open skull injury models while the weight drop impact acceleration (WDIA) model is representative of closed skull injury model, the effects of which are similar to those which occur in motor vehicle and sporting accidents. Diffuse and focal TBI have some overlapping behavioral deficits (Gaetz, 2004; VNI, 2007/2008; Helps et al., 2008; Faul et al., 2010; Risdall and Menon, 2011), but also differ in outcomes since focal injury also leads to brain lesions, neuro-degeneration, and behavior changes like epilepsy generally not associated with diffuse TBI.

During the phase of rapid acceleration-deceleration and brain rotation, shear-tensile forces can cause direct severing of axons, an effect referred to as primary axotomy (Corbo and Tripathi, 2004). Such direct severing of axons, causing them to retract and form a retraction ball, is only found in the most severe cases of TBI (Povlishock and Katz, 2005). More commonly, in mild and moderate forms of TBI there is secondary axonal swelling and disconnection, a process referred to as secondary axotomy (Pettus et al., 1994; Büki and Povlishock, 2006). Secondary axotomy appears due to several mechanisms (Farkas and Povlishock, 2007). One mechanism involves axonal swelling and subsequent axotomy due to the disruption by the primary insult of underlying axoplasmic cytoskeletal components (Maxwell et al., 1997; Farkas and Povlishock, 2007). As delivery of substances continues through normal transport kinetics in a disrupted cytoskeleton, intracellular protein and cellular organelles accumulate along sections of the axon, leading to progressive swelling and collapse several hours after initial injury (Kelley et al., 2006; Farkas and Povlishock, 2007; Greer et al., 2013). This accumulation makes it possible to microscopically identify DAI by axonal retraction bulbs. Other mechanisms of axonal disconnection include altered membrane permeability, activation of cysteine proteases, cytoskeleton breakdown and mitochondrial swelling. In this instance, DAI is not detected by hallmark neuropathological swollen bulbs, as there is a conversion of anterograde to retrograde transport which blocks the occurrence of swelling (Povlishock and Katz, 2005), but β-amyloid precursor protein, an indicator of impaired axonal transport, accumulates at the site of axotomy (Gentleman et al., 1993). After axonal severing, downstream axonal segments will undergo Wallerian degeneration, as early as 1–3 h after trauma, but even up to several months post-impact (Kelley et al., 2006).

The exact progression of these changes will vary between patients with injury type and severity, and patient-related factors. Knowledge of how these changes translate to the changes in neuronal information processing that cause long-term cognitive, motor and sensory deficits is critical for prognosis, to ensure therapy is appropriate for brain processes occurring *at that time* in each patient.

## Sensory Cortex as a Systems Neuroscience Test Bed

Our internal world and our responses to the external world are often driven by the input we receive of that external world. Thus, our underlying thesis is that many of the prolonged cognitive, sensory, movement and memory deficits after TBI are exacerbated from deficits in sensory processing. This hypothesis is framed in the context of the accumulating evidence in humans and animals that the prolonged cognitive, sensory, movement and memory deficits after TBI may flow-on from deficits in sensory processing (Strich, 1956; Bawden et al., 1985; Haaland et al., 1994; Gagnon et al., 1998; Graham et al., 2000; Draper and Ponsford, 2008, 2009; Ponsford et al., 2008; Little et al., 2010; Bayley et al., 2014), even in mild/moderate TBI (Strich, 1956; Bawden et al., 1985; Haaland et al., 1994; Gagnon et al., 1998; Graham et al., 2000; Draper and Ponsford, 2008; Little et al., 2010). It is also the case that sensory cortex is a good model system for studying post-TBI deficits in cortical processing because of its many features of topographic organization, its well-characterized neuronal responses to a wide range of sensory features, and the fact that sensory stimuli can be precisely quantified and reproduced between test sessions and animals.

To study the underlying neuronal functionality changes that underlie these deficits, we have conducted studies using an animal model, the rat barrel [somatosensory] cortex, a major sensory processor receiving tactile input from the face whiskers and guiding behaviors like perception, social interactions, navigation and guidance, and sensory and motor learning. The rodent whisker-recipient cortex (the postero-medial barrel sub-field (PMBSF) of somatosensory cortex the so-called “barrel” cortex) has been studied intensively over the past few decades owing to its distinctive structural and functional organization. It contains a highly organized somatotopic map of the rodent’s facial whiskers in the mystacial pad (Schubert et al., 2001). The mystacial pad system comprises short (microvibrissae) and long (macrovibrissae) whiskers arranged in an organized fashion along the snout in a grid-like pattern of arcs and rows (Woolsey and Van der Loos, 1970). Afferent information from each whisker projects via brainstem and thalamic nuclei to the PMBSF (details below).

Like other areas of the neocortex, neurons in PMBSF are arranged in layers that connect to different cortical and subcortical regions (Mountcastle, 1997). The neocortex comprises layers I-VI, with the cell-sparse layer I being the most superficial and layer VI the deepest and lying just above the white matter. In PMBSF, cells in the main thalamic input layer IV are clustered in a distinctive barrel-like manner (hence “barrel” cortex) with cells arrayed around a cell-sparse hollow. Each barrel receives input from a specific whisker, the Principal Whisker (PW; details below). The cells directly above and below each barrel represent a functional column extending from *supra* (layers I-III) to infragranular (V-VI) layers (Mountcastle, 1997). Barrels are separated by cell-sparse septa which are responsible for extra-columnar processing of information, and transfer of signal between adjacent barrels (Welker and Woolsey, 1974; Alloway, 2008). Afferent information from each whisker projects via brainstem and thalamic nuclei to an individual “barrel” in layer IV (Alloway, 2008) to define the PW of that barrel. Cells in a single functional column aligned with a specific barrel respond best to the same PW (Schubert et al., 2001) although they also respond to adjacent whiskers.

Input from the thalamus is relayed to granular layer IV, which mainly projects to layer II/III neurons, as well as other layer IV neurons. Supragranular neurons are responsible for integrating information both within a single functional column, as well as across adjacent cortical column, hence receiving inputs from other supragranular neurons, as well as pyramidal neurons from granular layer IV (Petersen, 2007, 2009). Output pathways for these supragranular neurons include projections to supragranular neurons in adjacent barrels, as well as projections to infragranular layers (LV; Welker et al., 1988; Hoeflinger et al., 1995; Petersen, 2007, 2009; Crochet and Petersen, 2009). Infragranular layers (Layers V and VI) are involved in output to other cortical areas such as secondary somatosensory cortex (S2) and the motor cortex (Fabri and Burton, 1991; Hoeflinger et al., 1995).

Three parallel afferent pathways carry tactile information from the whisker follicle to the cortex, with the pathways involving different brainstem and thalamic nuclei. The leminiscal pathway travels via the principal nucleus of the trigeminal and the dorsomedial section of the ventral posterior medial (VPM) nucleus of thalamus, to terminate densely in layer IV barrels of the PMBSF cortex. This pathway also has secondary projections that terminate in layers III, Vb, and VI in the same vertical column as the layer IV barrel. The paralemniscal pathway runs via the interpolar nucleus of the spinal trigeminal nucleus (subnucleus interpolaris; SpVi) and the medial part of the posterior medial (POm) to layers I and V (both barrel and septa but terminating most densely in the septa) of the PMBSF cortex and the S2 cortex. The secondary projections of this pathway terminate in layers II and III of the septal columns and in layers I and Va of both septal and barrel columns (Alloway, 2008). Finally, the extralemniscal pathway travels through the SpVi and the ventrolateral section of VPM to the septa between barrels in PMBSF cortex as well as to the secondary S2 (Alloway, 2008).

We now elaborate on the neuronal functionality changes in brain neurons after TBI, mainly focusing on the changes that we find in barrel cortex and the effects reported by others in hippocampus, and then suggest a putative mechanism to account for the effects. Our studies on barrel cortex neuronal functionality in the whole animal have provided the greatest amount of information on changes in brain neurons at the systems level, where studies at the level of the hippocampus, conducted in slices, have provided great understanding on changes in brain neurons at the level of synapses and channels. In the context of this review, our main focus will therefore be on the former set of data.

## Immediate and Long-Term TBI Effects on Cortical Neuronal Functionality

There are no studies of the continuous evolution of changes in neuronal functionality in the same animals and we describe here studies of neuronal functionality changes measured (in different studies) at discrete time points post-injury.

Studies of neuronal functionality in layer IV of barrel cortex after sustained cortical compression, a model of open skull TBI, found immediate suppression of neuronal activity, lasting for 5–20 min, followed by increased cortical activity by 2 h post injury (Ding et al., 2011). In other studies the hypoexcitation appears to be longer-lasting: metabolic studies show significantly reduced somatosensory circuit activation and depressed local cerebral metabolic rates of glucose even at 4 and 24 h after trauma (Dietrich et al., 1994).

**Figure 2:**
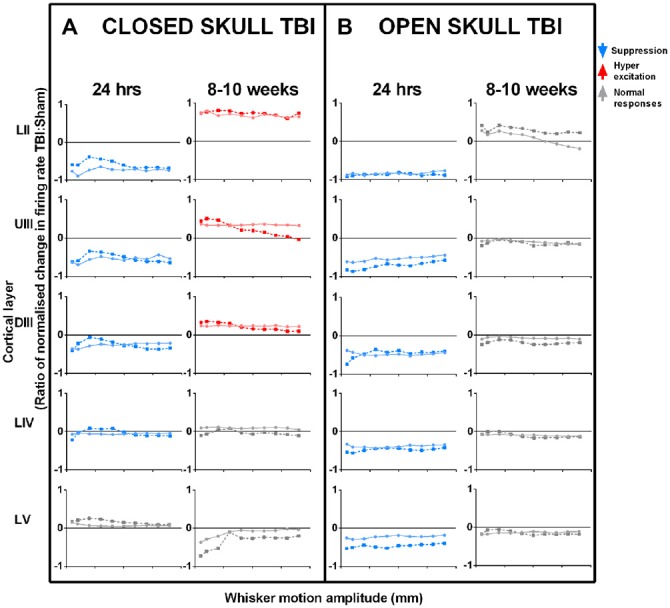
**Effects of TBI on sensory cortical neural responses in the short-term (24 h post-TBI) and in the long-term (8–10 weeks post-TBI).** Left columns **(A)**: closed skull TBI; (Alwis et al., 2012; Johnstone et al., 2013) Right columns **(B)**: open skull TBI (Johnstone et al., 2014, 2015). For both types of TBI, data are presented for effects seen 24 h post-TBI and 8–10 weeks post-TBI. In both models of TBI, immediate post-injury (24 h) effects of TBI are a suppression of responses, greatest in upper layers and decreasing with cortical depth. However, long-term effects (8–10 weeks) differ: in closed skull TBI there is Hyperexcitation in upper layers with all other layers showing normal responses, whereas mixed TBI results in normal responses in all layers. Data are neural response rates in TBI relative to Sham control, to two complex whisker motion waveforms (Ritt Rough and the Hartmann) as represented by the dotted and dashed lines. Data from top to bottom by depth of cortical layer: Layer II (LII), Upper Layer III (UIII), Deep Layer III (DIII), Layer IV (LIV) and Layer V (LV) (Data pooled across neurons in a layer; error bars omitted for clarity).

We examined the immediate post-TBI neuronal responses at a longer post-injury interval of 24 h post-injury. We examined effects for two major models of TBI with similar levels of severity—the weight-drop impact-acceleration model for severe diffuse injury or closed-head injury, and the lateral FP injury model for severe mixed diffuse and focal injury or open-skull injury. The effects seen in our studies are summarized in Figure 2 which plots the ratio of normalized change in neuronal firing rates in TBI and Sham control animals (Figure 2A: closed skull model TBI; Figure 2B: open skull model TBI). Responses were collected at different amplitudes of two complex whisker waveforms (Johnstone et al., 2014), from all neurons within a cortical layer, from Layer II through to Layer V from the somatosensory barrel cortex using a micro electrode (2–4 MOhm; FHC). Refer Supplementary Table 1 for sample sizes for each cortical layer for the two different models of TBI (Closed and Open skull) at the two different time points (24 h and 8–10 weeks). We recorded from neuronal clusters, of anesthesized animals to which we applied spike-sorting algorithms (Alwis et al., 2012) to extract single neuron data, to find that the effects were identical at single unit and population level, although amplified in the latter.

In both models, at 24 h post TBI there is a suppression of responses in the upper layers (compare first and third data columns in Figure 2: Layer II and Upper III, LII and UIII) and the effect decreases with cortical depth. This was true across simple and complex stimuli, and all types of excitatory cortical neurons (not shown). Changes in cortical activity occurring immediately post trauma are linked to the initial impact of the injury itself (Ordek et al., 2014) and involve elevation in intracranial pressure, damage to blood vessels and tissues, and axotomy, further inducing ionic imbalances (Ding et al., 2011; Johnstone et al., 2013). We have proposed (Johnstone et al., 2014) that this similarity occurs because of effects triggered by a stress wave initiated by the injury process, whether penetrating (in open skull TBI models) or not (in closed skull TBI models). In the open skull TBI model, we found that varying the distance of the injury site from 1–6 mm from barrel cortex did not change the depth-dependency (not shown), confirming that a remote spreading event caused the neuronal dysfunction. This factor is likely to be a wave of cortical spreading depression (CSD), which is characterized by rapid and almost complete depolarization of large populations of neurons, and propagates in the brain as a regenerating wave (Charles and Brennan, 2009). The wave spreads at a few mm/minute (Haaland et al., 1994; Charles and Brennan, 2009) with its leading edge in the upper layers which contain apical dendrites (Aitken et al., 1998). If depolarization persists, neurons enter an “unresponsive” state and can be rendered hypoactive in the long-term (Basarsky et al., 1998) through synaptic events such as long-term depression (Somjen, 2001). Cortical waves of depression have been observed after injury due to predominantly open skull injury models, including the open skull model (Katayama et al., 1990; Herreras and Somjen, 1993; Basarsky et al., 1998; Theriot et al., 2012) we use, but have yet to be documented after closed skull injury. Overall, our findings suggest CSD may likely be an important factor causing persistent cortical cellular changes after TBI, induced by either closed or open skull injury.

In the longer-term effects, hyperexcitability was reported in neocortical brain slices in layer V at 2 weeks CCI injury, another open skull model TBI (Yang et al., 2010). In our studies we found that the presence or otherwise of long-term hyperexcitation may depend on injury model. Thus, in contrast to the similar short-term effects described above, there were marked differences in long-term effects in the two forms of TBI we have studied. In closed skull TBI (Figure 2 2nd and 4th data columns), there was neuronal hyper-excitation in responses in the upper sensory cortical layers (Layer II, LII, and Upper III, UIII), normal responses in input layer (Deep III, DIII, and Layer IV, LIV; indicating normal sub-cortical inputs) and weak suppression in infra-granular Layer V neurons (LV). The hyper-excitation in upper layers is consistent with the fact that brain injury selectively affects inhibitory neurons (Cantu et al., 2015). This will change the excitation:inhibition (E:I) balance to favor excitation, causing hyper-excitation, and is discussed more fully below. Short term changes in excitatory transmission can also occur (Faden et al., 1989; D’Ambrosio et al., 1998; Sick et al., 1998; Witgen et al., 2005; Norris and Scheff, 2009), but the long-term hyperexcitability (Alwis et al., 2012) is more simply explained by changes in inhibition, of the type recently demonstrated by Cantu et al. (2015), which indicates that excitation is enhanced due to loss of inhibitory control following a CCI injury, due to a loss of inhibitory neurons in the TBI cortex.

As noted above, neurons in LII and LIII receive excitatory input mainly from LIV (Feldmeyer et al., 1999, 2002, 2006; Lübke et al., 2000; Schubert et al., 2001, 2007; Shepherd and Svoboda, 2005; Lübke and Feldmeyer, 2007; Alloway, 2008; Lefort et al., 2009; Hooks et al., 2011). Since they integrate responses across barrel cortex and output to other cortical areas, changes in LII and UIII are likely to contribute significantly in TBI to deficits in cognition and complex behaviors dependent on sensory input. The effect of LII and UIII changes can be seen even within the same column: in diffuse TBI, LV (with inhibition from LII/UIII) shows opposite effects to upper layers, whereas in mixed focal and diffuse TBI, LV shows the same effects as seen in the upper layers, and this likely results in sub-cortical changes that are different to the intra-cortical effects due to upper layer changes. Also we have previously reported that in the long term post-trauma cases where we find hyper-excitability in Layers II and Upper III, input layer IV of the same cortex shows perfectly normal responses in times of peak firing rates, excitatory responses over the entire stimulus period, the latency to the peak firing rate and the temporal dispersion of responses. Since layer IV predominantly receives the thalamic input to cortex, and its responses reflect these inputs, these data strongly suggest that sub-cortical regions appear to be normal in our diffuse TBI model.

In the open skull model TBI (Johnstone et al., 2015), “normal” long term responses occurred in all cortical layers (Figure 2 4th data column) despite major structural changes (Johnstone et al., 2015) which did not occur in the closed skull TBI model (Yan et al., 2013). We believe that the difference in long-term neuronal and structural changes in the two forms of TBI (closed and open skull) must account for different behavior outcomes (Hallam et al., 2004) as evidenced in the long term after closed skull TBI, diffuse injury in particular, where TBI animals showed persistent sensorimotor deficits up to 6 weeks post trauma and this was particularly evident in tasks related to direct whisker sensory processing (Alwis et al., 2012) while the open skull injury model showed cognitive and motor deficits and heightened anxiety-like behavior even up to 12 weeks after trauma (Johnstone et al., 2015). Open skull injury animals also showed greater impairment in memory tasks as opposed to closed skull injured animals (Hallam et al., 2004). The effect of LII and UIII long term changes can be seen even within the same column after closed skull diffuse TBI, LV (with increased inhibition from LII/UIII) shows opposite effects to upper layers (LII and UIII), whereas in long term open skull mixed diffuse and focal TBI, LV shows the same effects as seen in the upper layers (LII and UIII), and this effect in LV must result in sub-cortical changes that are also different to the intra-cortical effects due to upper layer changes. Our data support the theory that cortical supragranular layers 2/3 maintain their roles as “privileged substrates” (Nichols et al., 2007) for cortical plasticity. This could explain why TBI in both models produces changes in neuronal responsiveness in L2/3 where excitatory feedback is likely to amplify input from granular layer IV. Neuronal activity as indicated by cFos activation was attenuated even a week after closed skull TBI but then increased above sham levels by 4 weeks after trauma (Hall and Lifshitz, 2010). Consistent with this increased cFos activation in the longer-term, our electrophysiological studies showed hyperexcitation in the supragranular barrel cortex at 8–10 weeks post closed skull TBI (Alwis et al., 2012), as noted above. Cortical short-term hypoactivity transitioning to long-term hyperexcitation after closed skull brain injury is suggestive of initial circuit disruption followed by delayed cortical reorganization. Maladaptive circuit reorganization is a putative mechanism that could result in excitation/inhibition imbalance (Alwis et al., 2012, 2013; Greer et al., 2013).

**Figure 3:**
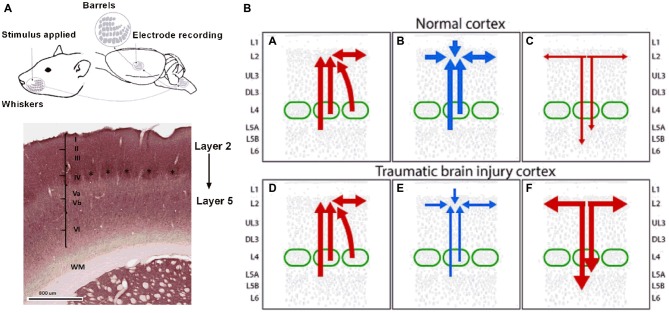
**Putative alterations in inhibitory circuits in supragranular layers 2/3 following the long term after diffuse TBI.** Panel **(A)** on the left shows a schematic representation of the rat whisker tactile system and electrophysiology recording from the barrel cortex. Evoked extracellular neuronal responses were recorded from the somatosensory barrel cortical layers 2–5 in response to whisker stimulation. Note the cluster of cells or barrels as denoted by “*” in cortical layer 4. Panel **(B)** on the right shows hyperexcitation in the supragranular layer 2 and Upper 3 in the long term after diffuse TBI resulting from either reduced local L2/U3 inhibitory inputs or due to a loss of excitatory drive from L2/U3 to deeper infrangranular layers which feed inhibition from L5 to L2/3. Top Row: all panels show circuitry in normal cortex, Lower Row: all panels show circuitry in TBI cortex. Left Panels **(A,D)** show excitatory inputs (in red) to L2/3 from local L2/3, L4 and L5 excitatory pyramidal neurons within and neighboring cortical columns. Panel **(B)** shows inhibitory inputs (in blue) to L2/3 from local L2/3 inhibitory neurons, input layer L4 and deeper L5. Panel **(E)** shows diminished inhibitory input as represented by the thinner blue lines and arrows to L2/3 from L2/3, L4 and L5 in the TBI cortex resulting in hyperexcitabilty in L2 and U3. Right panels show excitatory outflow in normal **(C)** and hyperexcitable TBI cortex as represented by thicker red lines and arrows **(F)**. Figure details based on information in Xu and Callaway (2009) and Petersen and Crochet (2013).

While it is important to recognize that excitation can also be affected in the TBI brain (Faden et al., 1989; D’Ambrosio et al., 1998; Sick et al., 1998; Witgen et al., 2005; Norris and Scheff, 2009), loss of inhibition is the most likely change accounting for the neuronal hyperexcitability we reported to occur in sensory cortex after diffuse TBI. This review of inhibitory changes in TBI should be viewed therefore in the context that excitatory changes also occur and together with the newer findings of changes in inhibition, lead to a much better picture of the complex and dynamic neuronal functionality changes in the brain. We note that in keeping with these postulates, excitatory activity and connectivity is enhanced while inhibition is compromised after trauma (Bonislawski et al., 2007; Hall and Lifshitz, 2010; Alwis et al., 2012). We propose that in long term after closed skull TBI, hyperexcitation in the supragranular Layer II and Upper III is due to loss of inhibition in these layers either due to reduced local intra-LII/UIII inhibitory inputs to local excitatory neurons or due to a loss of excitatory drive from LII/UIII to deeper infragranular inhibitory neurons such as the Martinotti cells (MC) in LVA which feedback inhibition to LII/UIII excitatory neurons and other interneurons. This hypothesis is elaborated schematically in Figure 3, which shows specifically the barrel cortex circuitry relevant to LII/UIII. This proposal is consistent with the fact that in the short-term, there is a depth-dependency to the electrophysiological changes in barrel cortex in both closed-skull and open-skull models of injury (Alwis et al., 2012, 2013; Johnstone et al., 2013, 2014, 2015; Yan et al., 2013).

Histologically, neuroplasticity markers such as growth associated protein (GAP-43) and synaptophysin, a pre-synaptic regenerative marker were elevated above sham controls as early as 3 days (Hulsebosch et al., 1998) and at 4 weeks post injury in the hippocampus suggestive of dynamic circuitry reorganization through synaptogenesis and regeneration (Hall and Lifshitz, 2010).

## Loss of Inhibition Underlies TBI and Many Other Brain Pathologies

Under normal conditions, the activity of cortical neurons is a finely-balanced interplay between excitation and inhibition (E and I) and a balance between these two opposing synaptic conductances is essential for proper cortical function. Then, manipulations in animal models of experimental TBI that selectively decrease either excitation or inhibition will shift cortical activity to result in either a hypoexcitatable or hyperexcitable state (Dudek and Sutula, 2007). There is accumulating evidence that changes in inhibition appear to be one of the major changes that modulates the E:I balance in the long-term TBI brain, as in other brain disorders, as we will review here.

### Diversity and Classification of Cortical Inhibitory Neurons

The neocortex contains a diversity of cell types, 20–30% of which are inhibitory neurons (that release the neurotransmitter GABA) with diverse morphological, molecular and electrophysiological properties (DeFelipe, 1993; Thomson and Deuchars, 1994; Cauli et al., 1997; Kawaguchi and Kubota, 1997; Somogyi et al., 1998; Gupta et al., 2000) and therefore it is possible to classify them on the basis of these characteristics. They can be clearly distinguished from excitatory neurons in that most mature inhibitory neurons have aspiny dendrites.

The axonal arborizations of inhibitory neurons are usually confined to their respective cortical columns but can also extend horizontally across columns but do not project to white matter areas (DeFelipe, 2002). Interneurons are arranged in highly ordered circuits within the cortical layers, and also connect to different cortical and subcortical regions, allowing for signal modification throughout the cortical and subcortical network. The interactions between excitatory pyramidal cells and GABAergic interneurons fall into two broad categories namely: “feedback” inhibition, in which an inhibitory neuron synapses back onto the same excitatory neuron which activated it and provides negative feedback and “feedforward” inhibition in which excitatory neurons synapse onto inhibitory neurons which do not synapse back onto those same excitatory neurons. Feedback and feedforward inhibition form the two fundamental building blocks of cortical inhibition (Isaacson and Scanziani, 2011) and both might be altered following injury.

Various methods are used to identify and differentiate between the subtypes of cortical inhibitory neurons including their firing properties, cortical location, morphology and immunochemistry (Kawaguchi and Kubota, 1997; Gupta et al., 2000; Markram et al., 2004). One major means used to discriminate between the different interneuronal subtypes is by their differential expression of neuromarkers such as calcium binding proteins [calbindin (CB), parvalbumin (PV) and calretinin (CR)] or neuropeptides [neuropeptide Y (NPY), Somatostatin (SOM), vasointestinal peptide (VIP), neuronal nitric oxide synthase (nNOS) and cholecystokinin (CCK)] or combinations of both. The heterogenous GABAergic interneurons have specialized functions in regulating excitatory neuronal activity by innervating a specific subdomain (soma, axon and dendritic regions) of the pyramidal cell (DeFelipe, 1997; Somogyi et al., 1998; Markram et al., 2004; Refer Table 1). For example, Chandelier cells (ChCs) that express the calcium binding protein PV uniquely target the axon initial segment of excitatory pyramidal neurons (Inda et al., 2009) and are capable of suppressing the initiation of action potentials in pyramidal neurons (Miles et al., 1996), acting as “rectifiers” of local circuit activity (Zhu et al., 2004) when network excitability goes out of control.

**Table 1:** **Differential neurochemical expression, innervation type and function of major inhibitory neuronal subtypes**.

Inhibitory neuronal subtype	Neurochemical marker	Target membrane domain	Function
**Chandelier cells**	PV or CB	Axon initial segment	Edit a neuron’s output by affecting generation and timing of action potentials (APs)
**Basket cells**			
• Large	PV, CB and NPY, cholecystokinin (CCK) occasionally SOM and CR	Soma and proximal dendrites	Allows presynaptic neurons to control the gain of summated potentials and thereby control AP discharge of target cells—(phasing and synchronization of neural activity)
• Small	VIP		
• Nested	PV or CB		Affects the generation and propagation of dendritic calcium spikes
**Martinotti cells**	SOM	Distal dendrites and tufts	Affects the generation and propagation of dendritic calcium spikes
**Bitufted cells**	CB, CR, NPY, VIP, SOM or CCK	Dendrites	Influences dendritic processing and integration of synaptic inputs.
**Bipolar cells**	VIP	
**Double bouquet cells**	CB, CR and CB, VIP or CCK		Influences synaptic plasticity either locally or by integrating with back propagating APs
**Neurogliaform cells**	nNOS		

*Inhibitory neurons can alter a specific activity of the target cell (pyramidal neuron) by selectively innervating a specific membrane domain. (Table derived from information in Markram et al., 2004; Spruston, 2008)*.

Basket cells (BCs) that mostly express two calcium binding proteins (DeFelipe, 1997) PV and CB are characterized by their extensive axonal arborizations that exert inhibitory effects on neurons in *supra* and *infra* granular cortical layers in neighboring and distant columns and are therefore considered as a primary source of lateral inhibition(Kisvárday et al., 1993). Large number of interconnections between BCs also suggests their involvement in long range lateral inhibition (Kisvárday et al., 1993). These cells are also thought to be involved in the gamma oscillations which enable fast processing (Fries et al., 2007) required for high levels of cognitive control.

MC, another class of GABAergic interneurons predominantly express the neuropeptide, SOM (Wang et al., 2004; Gentet et al., 2012) and are capable of extensive horizontal axonal projections extending upto millimetres in length within cortical layer I (Kawaguchi and Kubota, 1997; Wang et al., 2002), thereby inhibiting the dendritic tufts of pyramidal cells in nearby and distant columns. MCs are considered to be the only source of cross columnar inhibition via layer I to layers II-VI (Wang et al., 2004).

The CB and CR immunoreactive (IR) groups of neurons include the dendrite targeting bipolar, bitufted cells (BTCs) and double bouquet cells. Bipolar and double bouquet cells mainly target the basal dendrites of pyramidal neurons (Markram et al., 2004) and are thought to be involved in regulating the activity of other cortical GABAergic neurons through disinhibition (Wang et al., 2002). BTCs on the other hand form inhibitory synaptic contacts by projecting their axon collaterals onto pyramidal cells, neighboring BTCs and multipolar cells within the same layer and influence synaptic plasticity through back propagation of dendritic action potentials (Kaiser et al., 2001) while VIP IR neurons comprise of cells with radially oriented dendrites that extend to several cortical layers and are involved in translaminar inhibition (Kawaguchi and Kubota, 1996; Cauli et al., 1997; Bayraktar et al., 2000) and neuronal communication between pyramidal cells and other interneurons.

Other GABAergic neurons include neurogliaform cells (NGFCs) that form local horizontal axonal arborizations in layer I of the cortex (Chu et al., 2003) and are characterized by the expression of nNOS (Kubota et al., 2011). The multipolar Cajal Retzius cells are another class of interneurons found in layer I. A subset of these multipolar cells expresses the calcium binding protein CR in addition to alpha-actinin-2 (Aac; Kubota et al., 2011). The axons of these cells have extensive horizontal trajectories that target the terminal dendrites of pyramidal cells also mediating dendritic inhibition.

Thus owing to their diverse characteristics impairment of specific sub-populations of inhibitory neurons is likely to have different functional consequences and effects on cortical excitability and information processing.

### Changes in Inhibition in TBI

The neocortex and hippocampus are brain regions that appear particularly susceptible to TBI. Following TBI, they have been shown to undergo synaptic reorganization consistent with changes in the balance between excitation and inhibition.

#### Cortex

Spontaneous and evoked burst discharges have been reported in the rat neocortical layer V brain slices 2 weeks after CCI, an open skull injury, and postulated to be due to decreased inhibition (Yang et al., 2010). As noted above, Ding et al. (2011) reported that after open skull injury, there was hyperexcitability in the cortex 2 h post injury, the longest time point they examined. Such increases in cortical excitation following cortical injury (Imbrosci and Mittmann, 2011) are mainly due to loss or reduced activity of inhibitory neurons. Cortical hyperexcitability and elevated glutamate activity are associated with increases in frequency and amplitude of spontaneous excitatory synaptic currents and a decrease in frequency of spontaneous inhibitory synaptic currents 2–6 weeks in the chronically injured epileptogenic neocortex (Li and Prince, 2002). This was also observed in cortex after an open skull CCI brain injury, accompanied by reductions in the number of PV and SOM interneurons at 2–4 weeks following injury (Cantu et al., 2015). Loss of GABAergic control over pyramidal neurons would produce cortical pyramidal cell hyperexcitability but does not have to involve loss of the GABAergic neurons. Indeed, following CCI injury there were no changes in number of NeuN positive cells in the injured cortex of TBI animals compared to sham animals, but the density of PV- and SOM-IR inhibitory neurons was decreased in the injured cortex at 2–4 weeks post-injury (Cantu et al., 2015). In a similar vein we have postulated that in the closed-skull TBI model we have used, the long-term hyperexcitability results from a reduction in certain subsets of GABAergic interneurons or in their activity rather than a complete loss of inhibition (Alwis et al., 2013).

The postulated consequences to circuitry balance and function of loss of different forms of inhibition is summarized in Table 2. Note that the effects can and do differ for different brain regions since GABAergic interneurons likely have specific local functions in each site. Thus, there is a diversity of effects reported, but the overwhelming effect is of hyperexcitation. SOM-IR interneurons appear to be particularly efficient in counteracting increasing levels of cortical excitability and a selective loss of these dendrite targeting cells has been reported in experimental animal epilepsy models (Cossart et al., 2001) and human patients (de Lanerolle et al., 1989). This suggests the involvement of these interneurons in generation of epileptic seizures. Chandelier cells, that predominantly express the calcium binding protein PV and specifically innervate the axon initial segment of pyramidal cells, are reported to be strongly involved in preventing hyperexcitability in the cortex (Zhu et al., 2004) and a selective loss of these cell types in epileptic loci is indicative of their involvement in epileptic activity (Ribak, 1985). Human TBI studies by Buriticá et al. (2009) reported a decrease in PV-IR inhibitory neurons in layer II, and increases in interneuron-targeting CB-IR in layers III and V and CR-IR in layer II of the cortex. Finally, the functional implication of a loss of PV expressing interneurons following TBI like that reported by Pavlov et al. (2011) is deficits in gamma oscillations and impaired modulation of excitatory signals (Sohal et al., 2009).

**Table 2:** **Putative alterations in circuitry balance and function as a consequence of changes in the number of inhibitory neurons following TBI and epileptic seizures**.

Interneuron Subtype in function	Putative alteration	
Parvalbumin	Impaired perisomatic inhibition (Huusko and Pitkänen, 2014) and reduction of miniature inhibitory post-synaptic currents (mIPSCs; Knopp et al., 2008)
	Loss of long range inhibition to adjacent cortical columns (Buriticá et al., 2009)
Calbindin	Hyperexcitability in Dentate gyral circuits and impaired dendritic inhibition of pyramidal cells (Maglóczky et al., 1997; Carter et al., 2008)
	Impaired columnar inhibition (Buriticá et al., 2009)
Calretinin	Impaired synchronization of dendritic inhibitory neurons.
	Inefficient control of excitatory inputs to pyramidal cells resulting in impaired synaptic plasticity and seizure generation (Toth et al., 2010)
Neuropeptide Y	Impaired dendritic inhibition (Huusko et al., 2015)
Somatostatin	Impaired dendritic projections to pyramidal cells resulting in hippocampal hyperexcitability and generation of epileptic seizures (Cossart et al., 2001).
Cholecystokinin	Impaired perisomatic inhibition (Huusko et al., 2015)

*Functional consequences of loss of particular subsets of Inhibitory neurons following trauma and epilepsy (Table derived from information in Maglóczky et al., 1997; Cossart et al., 2001; Carter et al., 2008; Knopp et al., 2008; Buriticá et al., 2009; Toth et al., 2010; Huusko et al., 2015)*.

Overall, it can be postulated that trauma induced reduction in the activity of certain subsets of interneurons could compromise the excitation/inhibition balance, while a loss or reduction in the functionality of inhibitory cells could have profound effects on cortical information processing.

#### Hippocampus and Thalamus

In keeping with these postulates, several studies have also reported reductions in the number of GABA receptors and/or GABAergic neurons in hippocampal sub regions of the hilus and dentate gyrus after different forms of TBI and experimental epilepsy (Lowenstein et al., 1992; Toth et al., 1997; Santhakumar et al., 2000; Cossart et al., 2001). Santhakumar et al. (2000) reported a decrease in hippocampal hilar interneuronal populations labeled with GAD67 and PV mRNA probes following an FP injury. Transient reductions were seen in hippocampal GABA_B1_ and GABA_B_ mRNA while irreversible reductions in GABA_B1_ and GABA_B2_ mRNA expression in thalamus ipsilateral to the injury were seen even up to 4 months post TBI (Drexel et al., 2015). Mild TBI caused no neuronal loss (Eakin and Miller, 2012; Almeida-Suhett et al., 2014). However in moderate TBI the number of GABAergic interneurons was significantly reduced in the CA1 region at 7 days following a CCI (Almeida-Suhett et al., 2015). Finally, in severe TBI, there are reductions in PV-IR interneurons in the ispsilateral and contralateral thalamic VPM-VPL complex (Huusko and Pitkänen, 2014) and substantial reductions in PV, CR, neuropeptide (NPY), CCK and SOM interneurons in hippocampal subfields CA1, CA3 and dentate gyrus (DG), even up to 6 months after open skull TBI (Huusko et al., 2015). A progressive loss in phasic inhibition associated with a loss in PV positive GABAergic neurons was reported in the ipsilateral and contralateral hippocampus following a LFPI, an open skull TBI (Pavlov et al., 2011). These various studies therefore suggest a graded loss of hippocampal inhibition, at least in particular brain regions, dependent on injury severity.

However, other studies report GABA upregulation in the long term after TBI, as a compensatory mechanism to counteract increases in glutamate network activity. Upregulation of GABA_A_ receptor subunits was seen in hippocampal sub-regions 10 days and/or 4 months after TBI (Drexel et al., 2015). Increases in GABAergic NPY expressing interneuron fibers have been reported in the injured parietal cortex following a FP model of TBI, and it was suggested this indicated a role of NPY neurons in neuroprotection (McIntosh and Ferriero, 1992).

Functional and circuit reorganization occur in the cortex and hippocampus following TBI. Glutamate excitotoxicity, a secondary injury mechanism of TBI, alters dendritic outgrowths in GABAergic cortical neurons independent of cell death (Bywood and Johnson, 2000). Morphological and biochemical alterations, such as dendritic regression thought to have an effect on neurotransmission, occur in surviving interneurons further impairing cortical functionality (Monnerie and Le Roux, 2007). Other mechanisms that impact on cortical and hippocampal excitability levels include mossy fiber sprouting in hippocampus following CCI injury (Hunt et al., 2009) and increases in excitatory synapses (Buckmaster et al., 2002) and excitatory input to cortical pyramidal neurons (Brill and Huguenard, 2010).

### Changes in Inhibition in other Brain Disorders

Impaired inhibition, shifting the balance towards increased excitation, has been observed in other forms of brain disorders including epilepsy, stroke and schizophrenia. In a kainic acid induced experimental model of epileptogenesis (Fritsch et al., 2009), there was extensive loss in the number of GABAergic interneurons IR for GAD67. There was also a reduction in GluK1 sub unit, which is activated by glutamate to release GABA, and this would further contribute to a reduction of *tonic* inhibition in the basolateral amygdala (BLA) circuit. This was accompanied by increased protein levels of GAD65/67 expression and α1 subunit of GABA_A_ receptor at 7–10 days after status epilepticus (SE) in surviving interneurons presumably a compensatory effect for inhibitory neuronal loss (Fritsch et al., 2009). In the long-term after SE, ipsilateral reduction in *phasic* GABA_A_ mediated inhibition have been reported 1 month after TBI and a further significant decrease in synaptic inhibition in both hemispheres 6 months after TBI in a model of post-traumatic epilepsy (Pavlov et al., 2011). Reductions have also been observed in other GABAergic neurons such as CB expressing interneurons in the hippocampal DG of human epileptic patients (Maglóczky et al., 1997) and in animal models (Carter et al., 2008) of long term acquired epilepsy. Thus hippocampal CB neurons may be involved in hyperexcitability in the epileptic DG.

In both trauma and cerebral ischemic stroke, similar processes such as excitotoxicity, oxidative stress, inflammation and apoptosis contribute to loss of cell and tissue integrity. Degeneration of hippocampal GABAergic BCs occurs 12 h after cerebral ischemia (Crain et al., 1988) suggesting the selective vulnerability of inhibitory neurons in stroke just as in trauma. In cortex, accumulating evidence also indicates an excitation/inhibition imbalance in experimental models of stroke (Clarkson and Carmichael, 2009; Clarkson et al., 2010, 2011). Immediately after stroke in the first 30 mins within onset of reperfusion there is a rapid decrease in the expression of GABA_A_ receptor subunit (α1 and β2) mRNAs in hippocampal areas CA1, CA3 and DG (Li et al., 1993) accompanied by GABA_A_ receptors down regulation in cortex and hippocampus (Alicke and Schwartz-Bloom, 1995) which return to normal within 2 h post stroke. Cortical GABA levels are reduced in the short term, 8–18 days and in the long term, 6 weeks after stroke (Liepert et al., 2000; Bütefisch et al., 2008). Blicher et al. (2015) also found GABA levels to be reduced in the primary motor cortex in the long term, 3–12 months after stroke. Such reductions in GABA mediated inhibition facilitate functional recovery during periods of plasticity (Paik and Yang, 2014). Contrary to the above findings, GABA_A_ receptor mediated *tonic inhibition* increases significantly 3, 7 and 14 days after stroke due to a decrease in the normal uptake of extracellular GABA through neuronal and astrocytic GABA transporters (GAT; Clarkson et al., 2010; Carmichael, 2012). However, Alicke and Schwartz-Bloom found that during transient ischemia prolonged exposure to *in vitro* concentrations of synaptic GABA agonists could downregulate GABA_A_ receptors (Alicke and Schwartz-Bloom, 1995) by restoring the excitation/inhibition balance and promoting functional recovery. The persistent increase in tonic inhibition for up to 2 weeks following stroke is considered to be a compensatory mechanism of the brain to counteract and minimize neuronal injury.

Besides impaired GABA mediated inhibition following stroke, reductions in inhibitory CB expressing interneurons have also been reported in ischemic regions of cerebral cortex after middle cerebral artery occlusion (MCAO; Ouh et al., 2013). Transient cerebral ischemia has been reported to alter dendritic morphology of GABA_A_ receptor alpha 1-subunit-IR interneurons in CA1 of the hippocampus as early as 3 days persisting up to 5 weeks. Similar dendritic abnormalities have also been observed in CA1 interneurons even in long term, 12–14 months after ischemia (Arabadzisz and Freund, 1999) leading to altered GABA neurotransmission.

Like other brain disorders, schizophrenia has also been shown to be associated with multiple abnormalities in pre and post synaptic GABAergic PV expressing BCs weakening their inhibitory control over pyramidal cells (Lewis et al., 2012). Memory function is impaired in schizophrenia owing to diminished gamma-frequency synchronized neuronal activity resulting from altered *perisomatic inhibition* of pyramidal neurons (Lewis et al., 2005). A bilateral reduction in CB neuronal density was also reported in the planum temporale in schizophrenic patients, suggesting a reduction in *columnar/vertical inhibition* provided by double bond conversions (DBCs) that account for the majority of CB expressing interneurons in the brain (Chance et al., 2005).

Given that brain disorders, including TBI can alter inhibitory circuits in a number of ways and that inhibitory neurons play an important role in regulating cortical activity, our working hypothesis is that diffuse TBI can alter intra-cortical inhibition following diffuse TBI. We hypothesize further that altered inhibition, along with DAI, explains the pattern of responses we observed in the closed skull, diffuse injury model, and the open skull, mixed diffuse and focal injury model. Although the lack of detailed information regarding the timing and extent of post TBI changes in cortical cellular physiology make it difficult to speculate precisely, we argue broadly that in moderate-to-severe TBI, the initial stress wave from head or brain impact results in DAI which reduces responses at 24 h in both models; then over the next 8–10 weeks, additional, cell death from direct focal injury will result in slower death of excitatory and inhibitory neurons, dampening overall activity and returning the E:I balance to normal levels. However structural damage associated with focal injury will produce different deficits in the open skull model (diffuse and focal injury) compared to the closed skull model (diffuse injury only). Further, interplay between the effects of diffuse TBI [e.g., immediate axonal injury and long term interneuron damage] and focal injury (e.g., cell death) causes neuronal and behavior deficits to evolve more slowly in mixed (focal and diffuse) TBI.

## Conclusions for Future Directions

As we noted at the outset of the review, little has been (and is still) known of the changes in systems-level neuronal processing that cause long-term cognitive, motor and sensory deficits. There is a wealth of data on hippocampal electrophysiology at the slice level, but very little from the cortex, especially at the whole animal level. Our thesis is that prolonged cognitive and motor deficits in people with TBI may be due to sensory cortical deficits, induced by changes in the E:I balance. We have summarized the accumulating evidence that changes specifically in inhibition appear to be one of the major changes modulating the E:I balance in the long-term TBI brain, to favor excitation. The available evidence certainly shows that excitatory activity and connectivity is enhanced while inhibition is compromised after trauma, and may not be restricted to TBI alone amongst brain disorders. The existing data suggest that, at least in cortex, the major change is in the supragranular layers.

Against the relative uniformity of effects in cortex, at the sub-cortical level it has been reported that there is also upregulation of inhibition in long term TBI to counteract increases in glutamatergic activity. Thus, some regions of hippocampus show increased excitation but other regions show increased suppression in long-term TBI. There is sprouting of fibers in hippocampus and increases in excitatory synapses and excitatory input to cortical pyramidal neurons, all of which will impact on the EI balance in the brain.

With respect to neuronal functionality in the intact brain, our studies in the sensory barrel cortex of rats show that, at a systems level:

1. *Evoked neural activity* recorded with intra-cranial microelectrodes from neurons in upper cortical layers (those most accessible for sampling with extra-cranial electrodes) very reliably maps neuronal changes in TBI to behavior deficits [closed skull injury: (Alwis et al., 2012), open skull injury: (Johnstone et al., 2015)].
2. These neural changes are detected with microelectrode recordings even after *mild* TBI which shows no histological or molecular changes but results in mild behavior deficits.
3. Electrophysiology very well differentiates between *differences in the changes in neuronal functionality in different types of TBI*, which produce very similar neural changes in the short-term but very different long-term effects (and different behavior outcomes).

These data provide compelling evidence that electrophysiology can provide the high-precision information needed to monitor and understand the temporal evolution of changes in neuronal functionality in TBI. Non-invasive extra-cranial electrophysiological recording from the brain [using the electroencephalogram, (EEG)] is very attractive as the equipment is portable, the process is much more cost effective compared to any other method of monitoring brain activity, and testing can be done at the patient’s bedside. There has been a resurgence of interest in extra-cranial EEG recordings of neuronal activity in brain injury (Abend et al., 2010). However, electrophysiological recordings have been underutilized clinically and poorly studied and there is a dearth of basic and applied information on the evolution of changes in brain neuronal function and processing in different forms of TBI (Bosco et al., 2014). In fact a survey of 330 physicians concluded that “little data exists to allow for evidence based [continuous EEG] implementation or management related to [continuous EEG] findings” (Abend et al., 2010). Studies on the utility of quantitative EEG (qEEG) as a detection tool for mild TBI or concussion concluded that qEEG by itself provided limited diagnostic utility (Nuwer et al., 2005; Arciniegas, 2011; Rapp et al., 2015). Other review studies show the usefulness of continuous EEG and electrophysiological monitoring in the detection of seizures and SE (Schmitt and Dichter, 2015) and in estimating the prognosis of acute brain injury (Claassen and Vespa, 2014). Continuous EEG monitoring also allowed for identification and treatment of subclinical seizures in pediatric acute TBI patients (O’Neill et al., 2015). Other observational studies comparing the performance of quantitative handheld EEG to head CT provide growing evidence that it can be used to predict intercranial lesions in acute mild TBI (Ayaz et al., 2015). We believe that our data also provides a basis for such studies to now commence in humans. Our animal data show that driven activity is consistently altered in TBI, but differently long-term in various types of TBI. Hence, the use of evoked potentials in addition to the EEG will allow us to establish a successful biomarker for use in continuous, bedside monitoring of neuronal functionality in humans. Further, we have securely established that neuronal activity in upper cortical layers is a powerful indicator of cortical changes in TBI, and differentiates between different forms of TBI. The accessibility of these layers for non-invasive recording allows their activity to be monitored for the evolution of neuronal functionality changes with high-precision spatial and temporal definition.

We propose that monitoring of [resting] EEG activity as well as evoked potentials [e.g., visually evoked potentials (VEPs) and/or somatosensory evoked potentials (SEPs)] in human patients with moderate-to-severe TBI, at time periods from 24 h after admission to ICU post-TBI, through to 6 months post-TBI, would be invaluable in defining the evolution of neuronal functionality changes in TBI in each patient. As noted above, this has the potential to ensure therapy is appropriate for brain processes occurring *at that time* in each patient. Event related potentials and EEG spectral power have been used to identify deficits in response inhibition (Roche et al., 2004) with TBI causing changes in N2 and P3 waveform components in response to visual stimuli and changes in EEG alpha power (Roche et al., 2004). A recent exciting development in the use of EEG for bedside monitoring of TBI is the finding (Li et al., 2014) that novel EEG analysis technique of symmetrical channel EEG analysis (SESA) combined with sophisticated signal processing and statistical analyses of the approximate entropy (a measure of the regularity and unpredictability of signal fluctuations over time-series data such as the EEG) and the Slow Wave Coefficient (the ratio of the summed power in the delta and theta bands to the summed power in the alpha and beta bands, i.e., the ratio of the power in the low frequency bands to the power in the high frequency bands) well indexes unilateral TBI, the most common form of TBI. Other newer techniques are currently being developed and offer potential to distinguish severe TBI from mild and also TBI from other brain disorders (Tsirka et al., 2011; Prichep et al., 2012; McBride et al., 2013; Teel et al., 2014). We believe that the time is appropriate for clinical trials of the use of non-invasive extra-cranial electrophysiology to monitor and define the evolution of post-TBI changes in cortical neuronal functionality.

## Author Contributions

SFC—generated some part of the overall structure especially with respect to inhibition, wrote most of the manuscript and generated most of the figures. DSA—generated some part of the overall structure especially with respect to barrel cortex structure, wrote that section of the manuscript, assisted with editing, and generated Figure 1. RR—generated the overall structure of the manuscript, wrote and edited some part of the manuscript.

## Funding

The work represented here was funded by the National Health and Medical Research Council of Australia Project Grant No. APP1029311.

## Conflict of Interest Statement

The authors declare that the research was conducted in the absence of any commercial or financial relationships that could be construed as a potential conflict of interest.

## Supplementary Material

SUPPLEMENTARY TABLE 1 **Table summarizes sample sizes for each cortical layer for the two different models of TBI (Closed and Open skull) at the two different time points (24 h and 8–10 weeks) examined for two complex whisker deflection stimulus (Ritt Rough and the Hartmann)**.
