# Supplementary material for: Traumatic Brain Injury and Neuronal Functionality Changes in Sensory Cortex
Source: Front Syst Neurosci. 2016 Jun 2;10:47. doi: 10.3389/fnsys.2016.00047 (PMC4889613; doi:10.3389/fnsys.2016.00047)
Supplement: SUPPLEMENTARY TABLE 1 — Table summarizes sample sizes for each cortical layer for the two different models of TBI (Closed and Open skull) at the two different time points (24 h and 8–10 weeks) examined for two complex whisker deflection stimulus (Ritt Rough and the Hartmann). [file Table_1.docx]

| Closed Skull TBI | | | | |
| --- | --- | --- | --- | --- |
| 24 hrs | | | | |
| Layer | Ritt Rough | | Hartmann | |
| LII  UIII  DIII  LIV  LV | Sham  12  14  14  9  8 | TBI  8  10  10  14  13 | Sham  12  14  14  14  14 | TBI  7  9  10  9  8 |
| 8 weeks | | | | |
| Layer | Ritt Rough | | Hartmann | |
| LII  UIII  DIII  LIV  LV | Sham  12  12  12  12  10 | TBI  12  12  10  9  8 | Sham  13  11  10  10  7 | TBI  13  5  4  4  4 |
| Open Skull TBI | | | | |
| 24 hrs | | | | |
| Layer | Ritt Rough | | Hartmann | |
| LII  UIII  DIII  LIV  LV | Sham  12  14  14  14  13 | TBI  8  10  10  9  8 | Sham  12  14  14  14  14 | TBI  7  9  10  9  8 |
| 8 weeks | | | | |
| Layer | Ritt Rough | | Hartmann | |
| LII  UIII  DIII  LIV  LV | Sham  12  14  14  14  13 | TBI  7  10  10  9  8 | Sham  12  14  14  14  14 | TBI  7  8  10  9  8 |
